# Potent Anti-Inflammatory and Antiproliferative Effects of Gambogic Acid in a Rat Model of Antigen-Induced Arthritis

**DOI:** 10.1155/2014/195327

**Published:** 2014-01-30

**Authors:** Rita Cascão, Bruno Vidal, Helena Raquel, Ana Neves-Costa, Nuno Figueiredo, Vineet Gupta, João Eurico Fonseca, Luis Ferreira Moita

**Affiliations:** ^1^Instituto de Medicina Molecular, Faculdade de Medicina da Universidade de Lisboa, Lisbon, Portugal; ^2^Gulbenkian Programme for Advanced Medical Education, Lisbon, Portugal; ^3^Department of Internal Medicine, Rush University Medical Center, Chicago, IL 60612, USA; ^4^Rheumatology Department, Centro Hospitalar de Lisboa Norte, EPE, Hospital de Santa Maria, Lisbon, Portugal

## Abstract

*Background*. We have previously reported a continuous activation of caspase-1 and increased interleukin (IL)-1**β** levels in early rheumatoid arthritis (RA). These observations raised the hypothesis that drugs targeting the IL-1**β** pathway, in addition to tumour necrosis factor (TNF), may be particularly effective for early RA treatment. We have recently identified gambogic acid as a promising therapeutic candidate to simultaneously block IL-1**β** and TNF secretion. Our main goal here was to investigate whether gambogic acid administration was able to attenuate inflammation in antigen-induced arthritis (AIA) rats. *Methods*. Gambogic acid was administered to AIA rats in the early and late phases of arthritis. The inflammatory score, ankle perimeter, and body weight were evaluated during the period of treatment. Rats were sacrificed after 19 days of disease progression and paw samples were collected for histological and immunohistochemical evaluation. *Results*. We found that inflammation in joints was significantly suppressed following gambogic acid administration. Histological and immunohistochemical evaluation of treated rats revealed normal joint structures with complete abrogation of the inflammatory infiltrate and cellular proliferation. *Conclusions*. Our results suggest that gambogic acid has significant anti-inflammatory properties and can possibly constitute a prototype anti-inflammatory drug with therapeutic efficacy in the treatment of inflammatory diseases such as RA.

## 1. Introduction

Rheumatoid arthritis (RA) is the most common disease of the inflammatory joint diseases, afflicting about 1% of the world population. The disease can have a very aggressive course and poor outcome as inferred by the analysis of its social impact (after 10 years of disease duration, more than 50% of RA patients are unable to perform professional activities) [1] and life expectancy diminishes 10 years due to disease activity and associated comorbidities [2]. RA is a chronic systemic inflammatory disease characterized by synovial hyperplasia caused by a large proliferative cellular infiltrate of leukocytes, high expression levels of proinflammatory cytokines, and consequent erosion of joint cartilage and bone. The therapeutic approach of RA has been revolutionized in the last decade with the discovery of specific targeted treatments. However, despite all available therapeutic options, RA remains a progressive, destructive, and debilitating disease with only 20% of patients reaching remission [3]. Anakinra, an antagonist of interleukin (IL)-1, was approved for RA treatment in the last decade. However, the real impact on disease activity has been shown in practice to be lower than what was anticipated from clinical trial results, casting doubts on the role of IL-1*β* as a therapeutic target [4]. Nonetheless, we have previously reported increased levels of IL-1*β* in very recent onset arthritis and in the synovial fluid of established RA patients [5]. This observation could be explained by the activation of caspase-1 that we also have observed both in early and established RA patients [6]. Therefore, it is possible that IL-1*β* plays an important role in early rather than late stages of the disease and that pathways regulating this cytokine, such as caspase-1 and NF-*κ*B activation, can potentially constitute promising therapeutic targets for specific drugs. The effect might be further boosted if an inhibitory effect on tumour necrosis factor (TNF) can also be achieved. Based on the results of a recent drug screen for compounds that simultaneously inhibit IL-1*β* and TNF secretion, we chose gambogic acid as a promising therapeutic candidate for the treatment of arthritis. Gambogic acid is a polyprenylated xanthone abundant in resin derived from *Garcinia hanburyi* and *G. Morella* and is used in Southeast Asia complementary and alternative medicine [7]. Recent studies showed that gambogic acid could inhibit the growth of a wide range of tumour cells [8]. Our aim in this study was to investigate whether gambogic acid administration was able to attenuate inflammation in a rat model of antigen-induced arthritis (AIA).

## 2. Materials and Methods

### 2.1. Ethics Statement

All experiments were approved by the Animal User and Ethical Committees at the Instituto de Medicina Molecular, according to the Portuguese law and the European recommendations.

### 2.2. Compounds

Gambogic acid was purchased from Santa Cruz Biotechnology (Santa Cruz, USA).

### 2.3. IL-1*β* and TNF Quantification

THP-1 cells were stimulated for 6 hours with 4% PFA-fixed DH5 *Escherichia coli *(*E. coli*) at a multiplicity of infection (MOI) of 20 bacterial cells per THP-1 cell, 1 hour after incubation with gambogic acid. Cell supernatants were collected and IL-1*β* and TNF cytokines were quantified by enzyme-linked immunosorbent assay (ELISA) (R&D systems, USA) according to the provider's instructions.

### 2.4. AIA Rat Model and Assessment of Arthritis

Female Wistar AIA rats were purchased from Charles River Laboratories International (MA, USA) and maintained under specific pathogen-free (SPF) conditions. Animals were inoculated under isoflurane anesthesia by subcutaneous injection of complete Freund's adjuvant (CFA) containing *Mycobacterium butyricum* in the rat right pad which leads to a profound systemic inflammation resulting in severe joint swelling and destruction. Gambogic acid was administrated at a dose of 4 *μ*g/g body weight every day [9]. Drugs and vehicle control (dimethyl sulfoxide, DMSO) were dissolved in normal saline solution and injected intraperitoneally to AIA rats after 4 days (early treatment group, *N* = 10) and after 11 days (late treatment group, *N* = 5) of disease induction, when arthritis was already present. Healthy nonarthritic (*N* = 10) and vehicle-injected (*N* = 10) animals were used as controls for comparison. The inflammatory score, ankle perimeter, and body weight were measured during the time of treatment. Inflammatory signs were evaluated through the counting of the score of each joint in a scale of 0–3 (0: absence, 1: erythema, 2: erythema and swelling, and 3: deformities and functional impairment). The total score of each animal was defined as the sum of the partial scores of each affected joint [10]. Rats were sacrificed after 19 days of disease evolution and paw samples were collected for histological and immunohistochemical evaluation.

### 2.5. Histology and Immunohistochemistry

For histopathological observation, paws, lungs, livers, kidneys, spleens, and pancreas samples were collected at the time of sacrifice. Samples were fixed immediately in 10% neutral buffered formalin solution and then dehydrated using increased ethanol concentrations (70%, 96%, and 100%). Paw samples, after being fixed, were also decalcified in 10% formic acid. Samples were next embedded in paraffin, sectioned, and stained with hematoxylin and eosin for morphological examination. Paws were also used for immunohistochemical staining with Ki67 antibody, a cellular proliferation marker. Tissue sections were incubated with primary antibody against rat polyclonal Ki67 (Abcam, UK) and with EnVision + (Dako, Denmark). Colour was developed in solution containing diaminobenzadine-tetrahydrochloride (Sigma, USA), 0.5% H_2_O_2_ in phosphate-buffered saline buffer (pH 7.6). Slides were counterstained with hematoxylin and mounted. All images were acquired using a Leica DM 2500 (Leica microsystems, Germany) microscope equipped with a colour camera. Data regarding the degree of proliferation of synovial cells was scored from 0–3 (0: fewer than three layers, 1: three to four layers, 2: five to six layers, and 3: more than six layers). Lymphoid cell infiltration was scored from 0–3 (0: none to diffuse infiltration, 1: lymphoid cell aggregate, 2: lymphoid follicles, and 3: lymphoid follicles with germinal center formation) [11].

### 2.6. Caspase-1 and NF-*κ*B Assay

THP1 (ATCC TIB-202) macrophage-like cell line and THP1/NF-*κ*B reporter cell line were cultured in R10-RPMI media 1640 supplemented with 10% (v/v) fetal bovine serum, 1% (v/v) penicillin-streptomycin, 1% (v/v) pyruvate, 1% (v/v) L-glutamine, 1% (v/v) nonessential amino acids, 1% (v/v) hepes buffer, and 2-mercaptoethanol to a final concentration of 0.05 M, as recommended by the American Tissue Culture Collection (ATCC). Cells were cultured at 250.000 cells/mL, incubated with 10 *μ*M of gambogic acid for 1 h at 37°C 5% CO_2_, and then stimulated with PFA-fixed *E. coli *(20 *E. coli* per cell) for 8 h and 24 h at 37°C 5% CO_2_. Simultaneously, nonstimulated negative control cells were also cultured at the same density as the stimulated population for comparison. Caspase-1 activity was measured in THP1 macrophage-like cell line using the Carboxyfluorescein FLICA Detection kit for Caspase Assay (Immunochemistry Technologies, LLC, USA) following the reagent instructions. Briefly, cells from the different assays were protected from light exposure while incubated for 1 hour at 37°C with 30X FLICA solution at a 1 : 30 ratio. NF-*κ*B activity was measured in THP1/NF-*κ*B reporter cell line. Lentiviral particles carrying a NF-*κ*B-responsive GFP-expressing reporter gene (Cignal Lenti Reporters, SABiosciences, USA) were used to infect THP-1 cells and to establish a stable cell line. All samples were analyzed by flow cytometry using a FACS Calibur (BD biosciences, USA). The data collected were further analyzed using FlowJo software (Tree Star Inc., USA).

### 2.7. Statistical Analysis

Statistical differences were determined with nonparametric Kruskal-Wallis and Mann-Whitney tests using GraphPad Prism (GraphPad, USA). Differences were considered statistically significant for *P* < 0.05.

## 3. Results

### 3.1. Gambogic Acid Reduces IL-1*β* and TNF Production

**Figure 1:**
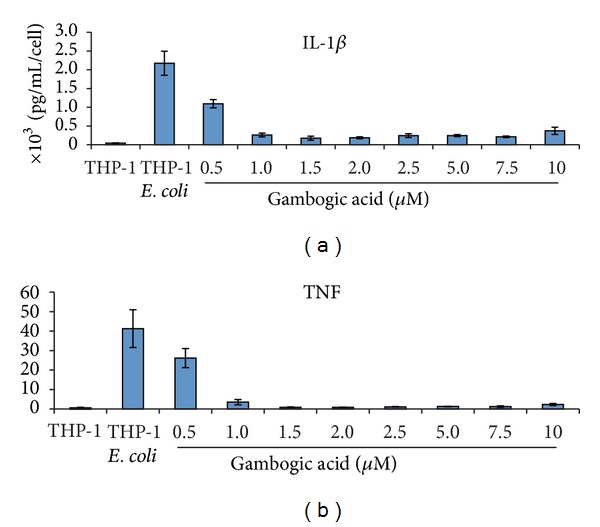
*IL-1*β* and TNF secretion are inhibited by gambogic acid treatment.* Media samples from human THP-1 macrophage-like cell line cultured with growing concentrations of gambogic acid were analyzed by ELISA technique. Differences were considered statistically significant for *P* values < 0.05.

To study the effect of this drug on the inhibition of IL-1*β* and TNF secretion, we treated the human THP-1 macrophage-like cell line with growing concentrations of gambogic acid for 1 hour before challenging them with PFA-fixed *E. coli* for 6 hours. The conditioned media was then probed for the secretion of either IL-1*β* or TNF using ELISA. Gambogic acid significantly inhibited the secretion of both cytokines over a wide range of concentrations (Figure 1), confirming the previously reported effect of gambogic acid in blocking the secretion of these cytokines [12] and validating our earlier findings.

### 3.2. Gambogic Acid Inhibits the Activation of NF-*κ*B and Caspase-1

**Figure 2:**
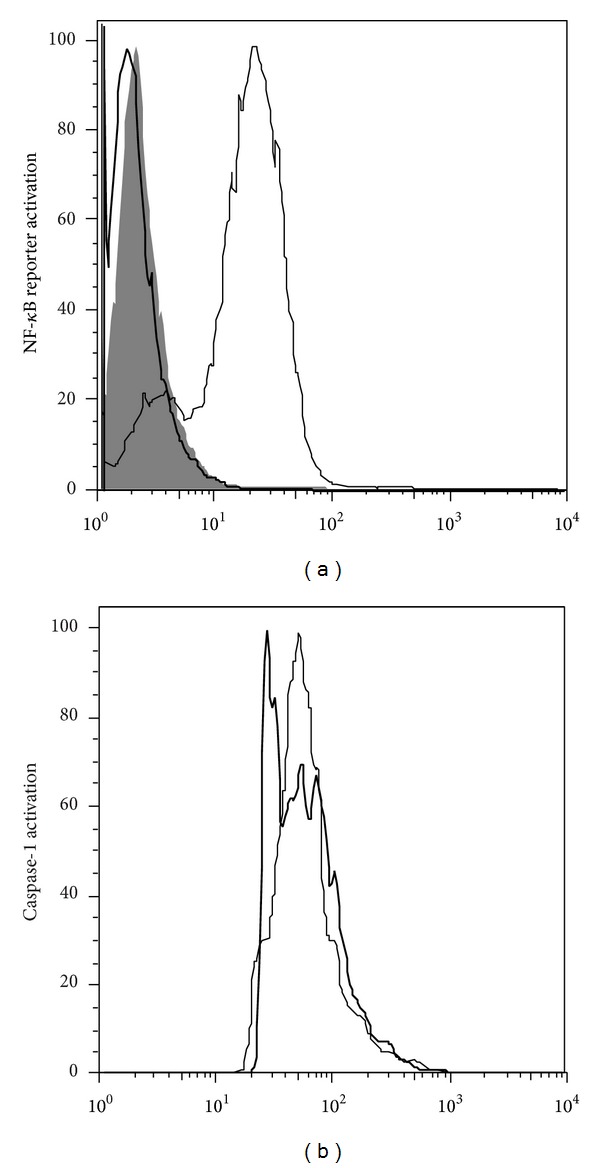
(a)* NF-*κ*B reporter activation is suppressed by gambogic acid treatment.* NF-*κ*B expression was measured by flow cytometry in a THP-1/NF-*κ*B reporter cell line incubated with gambogic acid and then stimulated for 24 h with *E. coli*. Each thin line in the histogram corresponds to untreated but *E. coli* stimulated cells, the shaded area corresponds to drug-treated and *E. coli* stimulated cells, and the thick line corresponds to untreated non-stimulated cells as a control. (b) *Caspase-1 activation is decreased with gambogic acid treatment. *Caspase-1 activation was measured using flow cytometry in a THP-1 cell line incubated with gambogic acid and then stimulated for 8 h with *E. coli*. Each thin line in the histogram corresponds to untreated but *E. coli* stimulated cells used as control and the thick line corresponds to drug-treated and *E. coli* stimulated cells.

Pro-IL-1*β* and TNF both depend on NF-*κ*B activation for the transcription of their respective mRNAs. Pro-IL-1*β* processing is further dependent on the activation of caspase-1. We therefore tested the effect of gambogic acid on these key pathways. To investigate the effect of this drug in the activation of NF-*κ*B, we used an NF-*κ*B reporter cell line created by stably infecting THP-1 cells with a commercial lentiviral GFP reporter under the control of a minimal CMV promoter and tandem repeats of the NF-*κ*B transcriptional response element (TRE). Gambogic acid was able to suppress NF*κ*-B reporter activation upon *E. coli* stimulation in comparison with cells that were also stimulated but did not receive treatment (Figure 2(a)). To test the effect of this drug on caspase-1 processing and activation, we used a caspase-1 fluorescent substrate and measured relative active caspase-1 levels using FACS. Also in this setting, gambogic acid significantly decreased the activation of caspase-1 (Figure 2(b)).

### 3.3. Gambogic Acid Is Able to Suppress Inflammation in Wistar Rat Antigen-Induced Arthritis

**Figure 3:**
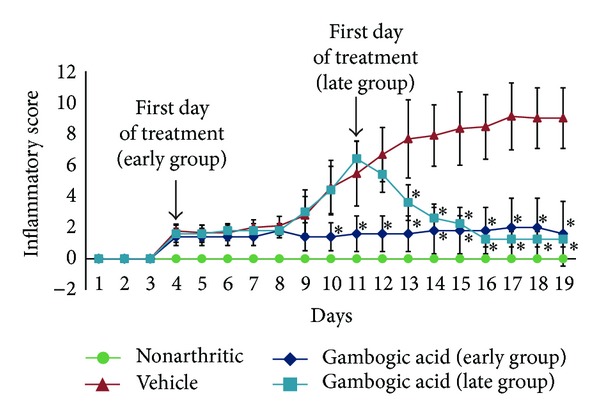
*Gambogic acid is able to suppress inflammation throughout time.* After 6 days of treatment, the vehicle-injected group increased inflammatory manifestations, whereas, in gambogic acid-treated rats, there was a significant reduction in the inflammatory activity. Arrows indicate the beginning of treatment after 4 and 11 days of disease induction. Differences were considered statistically significant for *P* values < 0.05.

**Figure 4:**
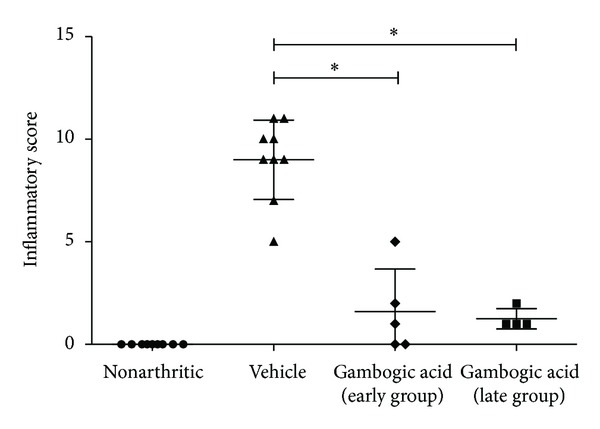
*Gambogic acid possesses anti-inflammatory properties.* Inflammatory score in gambogic acid-treated AIA rats is significantly diminished in comparison with vehicle-treated rats after treatment. Differences were considered statistically significant for *P* values < 0.05.

To study the anti-inflammatory properties of gambogic acid *in vivo*, AIA rats were treated daily with 4 *μ*g/g body weight of gambogic acid intraperitoneally after the disease had already become symptomatic. We started the treatment after 4 days of disease induction (early treatment group) and after 11 days of disease induction (late treatment group). The inflammatory score and ankle perimeter were evaluated during the period of treatment. As shown in Figure 3, by the 4th day, all induced animals already presented with arthritis. All induced animals received either vehicle or gambogic acid at that time point. After 6 days of treatment, the vehicle-injected group increased sharply the inflammatory manifestations, whereas, in gambogic acid-treated rats, there was minimal inflammatory activity or even complete abrogation of arthritis manifestations. In the late treatment group, drug administration was started after 11 days of disease evolution, when animals presented a mean inflammatory score of 6. Also in this group, by the second day of treatment with gambogic acid, the inflammatory manifestations started to significantly decrease over time. This result shows that this drug has anti-inflammatory effects even when administrated in a later phase of arthritis. After 15 (early treatment group) and 8 (late treatment group) days of treatment, gambogic acid showed significant anti-inflammatory effects, as assessed by the evaluation of the inflammatory score (Figure 4) and ankle perimeter (*P* = 0.0126 in early group and *P* = 0.0126 in late group versus untreated animals). We have also tested 2 *μ*g/g body weight subcutaneously every day. With this regimen, in the early treatment group, the anti-inflammatory effects were significant (*P* = 0.0048) but not as dramatic as the 4 *μ*g/g body weight intraperitoneally, and, in the late treatment group, the drug had no efficacy. In addition, we have observed that a subset of animals kept a low inflammatory score, even after stopping the administration of the drug at day 13. We were able to observe this anti-inflammatory effect in the absence of drug until day 21 (*data not shown*).

Of note, in some of the intraperitoneally treated animals, we have observed decreased body weight and ascites, in accordance with previous literature reports. During autopsy, we, together with a veterinary, have observed macroscopically internal organs that showed no alterations when comparing gambogic acid-treated, vehicle-treated, and healthy nonarthritic rats. Additionally, we have observed sections of internal organs collected at the time of sacrifice by histology, which have also shown no alterations comparing all experimental groups of animals. Specifically, in the case of the spleen, which in some RA patients and in animal models of arthritis present splenomegaly, we have observed that gambogic acid-treated and vehicle-treated rats showed spleen hyperplasia, with increased cellularity, compared with healthy nonarthritic rats (see Supplemental Figure S1 in Supplementary Material available online at http://dx.doi.org/10.1155/2014/195327). This result might be explained by the fact that gambogic acid is not modulating the lymphocyte development but is instead targeting downstream caspase-1 and NF-*κ*B activation.

### 3.4. Gambogic Acid Prevents Joint Inflammatory Infiltration and Proliferation

**Figure 5:**
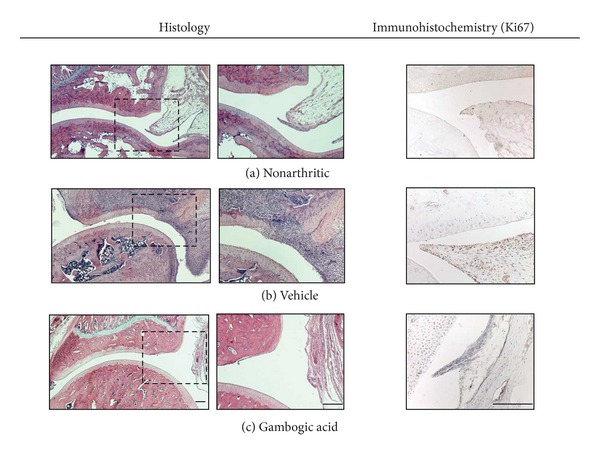
*Histological and immunohistochemical evaluation of joints after 15 days of treatment. *Nonarthritic control (a), vehicle-treated (b), and gambogic acid in the early treated group (c) AIA rats (magnification of 50x and 100x in histological images and a magnification of 200x in immunohistochemical images). Notice that gambogic acid has completely prevented immune cellular infiltration and proliferation, as well as bone invasion. Bars: 100 *μ*m.

To evaluate the infiltration of immune cells within joints in AIA rats, joint tissue sections were stained with hematoxylin and eosin. The histological evaluation shown in Figure 5 revealed that rats treated with gambogic acid had a normal joint structure with complete abrogation of the inflammatory infiltrate (*P* < 0.0001 in early treatment group versus untreated animals). In contrast, vehicle-treated rats exhibited infiltration of inflammatory cells, bone invasion and erosions (Figure 5). We also studied the levels of proliferation of immune cells by staining joint tissue sections with Ki67. The immunohistochemical results revealed that rats treated with gambogic acid presented reduced proliferation of immune cells within joints (*P* = 0.0098 versus untreated animals). Moreover, treatment with gambogic acid prevented cartilage and bone damage (Figure 5).

## 4. Discussion

Our results demonstrated that treatment with gambogic acid protected Wistar AIA rats from arthritis development with a complete abrogation of joint immune cellular infiltration and proliferation, preventing cartilage and bone damage.

Our laboratory has used a THP-1 macrophage-like cell line to screen 2320 drugs for those that simultaneously inhibit IL-1*β* and TNF secretion [13]. We have selected gambogic acid as a promising therapeutic candidate for the inhibition of both IL-1*β* and TNF secretion, due to the reduction in caspase-1 and NF-*κ*B activation, and consequently for the treatment of arthritis. Previous reports have demonstrated that gambogic acid can inhibit the growth of a wide variety of tumour cell lines, possibly due to its ability to induce apoptosis [14] via the transferrin receptor (TfR1) [15]. Additionally, recent data have shown that this drug can inhibit NF-*κ*B signalling pathway in human leukemia cancer cells [8] and in a noncancerigenous macrophagic cell line [16] also via TfR1. Therefore, the anti-inflammatory effects of gambogic acid appear to be mediated by the inhibition of NF-*κ*B activation pathway which in turn leads to the silencing of most of the inflammatory genes. In fact, the inhibition of NF-*κ*B in animal models has shown the ability to inhibit inflammatory arthritis, demonstrating that NF-*κ*B may be an important therapeutic target in RA [17–19]. Indeed, NF-*κ*B participates in the transcription of the genes encoding many proinflammatory cytokines and chemokines, in the regulation of the different immune cells, and in the regulation of the expression of adhesion molecules and matrix MMPs [20–22]. As recently reviewed, the IkB kinase IKK*β* is essential for the inflammatory cytokine-induced activation of NF-*κ*B [23]. Importantly, it has already been reported that gambogic acid is able to inhibit IKK*β* activity [16]. In our study, we demonstrated that the anti-inflammatory properties of this drug in AIA rats might not only be related with its ability to suppress the activation of NF-*κ*B but also to its effect on inhibiting caspase-1 activation.

Gambogic acid has a dual effect on the downregulation of TNF and IL-1*β* production. Interestingly, Joosten et al. showed uncoupled activities of IL-1*β* and TNF in joint swelling and ongoing cartilage destruction. Also, it has been reported that there are different time-dependent roles for IL-1*β* and TNF in the various stages of collagen-induced arthritis [24].

In RA, the inflamed synovium expands into and destroys the underlying cartilage and bone, resulting in irreversible erosion of the bone and in the loss of normal joint architecture leading to disability [25]. The inhibitory effect of gambogic acid in cellular infiltration and proliferation as well as in bone erosions can thus prove to be of interest to prevent and treat structural joint destruction induced by RA. In addition, administration of gambogic acid to AIA rats tested a therapeutic strategy, since it started in the early phase of arthritis, after 4 days of disease induction, with all animals already displaying signs of inflammation. To further evaluate the effect of the compound in a latter phase of the disease, gambogic acid was also administrated after 11 days of disease induction, when the animals displayed a high inflammatory score. Thus, gambogic acid treatment is effective when administrated both in the early and more established phase of arthritis, which is relevant to the possible clinical implications of our findings.

## 5. Conclusion

In conclusion, we have found that Wistar AIA rats can be effectively treated by gambogic acid. The effects of this drug probably rely on the inhibition of IL-1*β* and TNF secretion, possibly explained by its ability to downregulate caspase-1 and NF-*κ*B activation and by blocking synovial hyperplasia due to its significant antiproliferative properties. These results support our initial hypothesis that a double inhibition of IL-1*β* and TNF could be effective in the treatment of inflammatory diseases, such as RA, and further indicate that the antiproliferative properties of gambogic acid may prove essential for an effective clinical control and to induce early remission in RA patients. Based on our observations indicating possible toxicity in some of the regimens used, we suggest that gambogic acid might not be suitable to directly enter phase I clinical trials, but it can certainly serve as a prototype drug to search for derivate compounds with similar effects on inflammatory mediators and cell proliferation and a more favourable safety profile.

## Supplementary Material

We have observed macroscopically internal organs at the time of sacrifice as well as their histological sections, and concluded that there were no alterations comparing all experimental groups of animals. Specifically, in the case of the spleen, we have observed that gambogic acid treated and vehicle-treated rats showed spleen hyperplasia, with increased cellularity, compared with healthy nonarthritic rats.
