# Supplementary figures and images for: Potent Anti-Inflammatory and Antiproliferative Effects of Gambogic Acid in a Rat Model of Antigen-Induced Arthritis
Source: Mediators Inflamm. 2014 Jan 30;2014:195327. doi: 10.1155/2014/195327 (PMC3929289; doi:10.1155/2014/195327)

CONTROL

DMSO

GA

50X

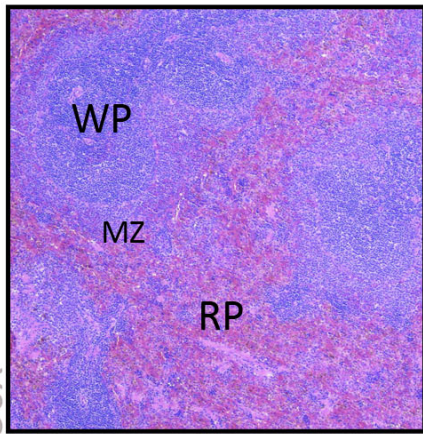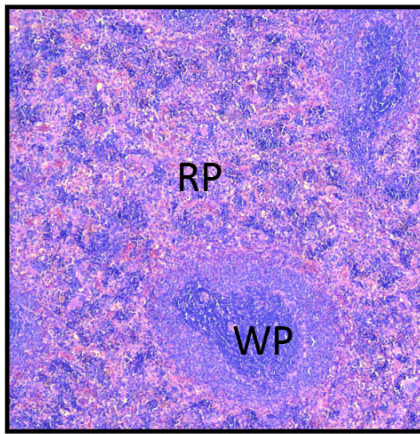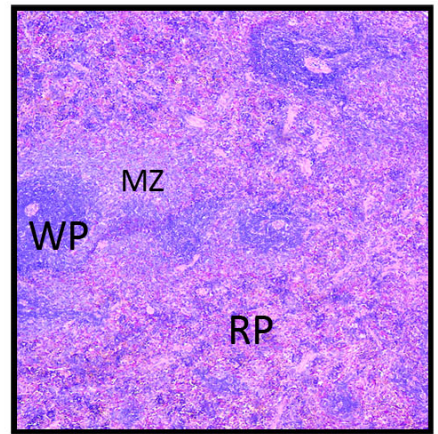

200X

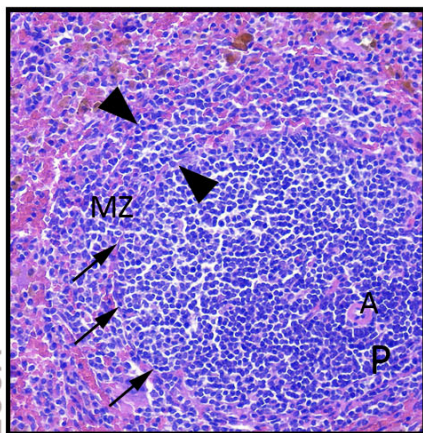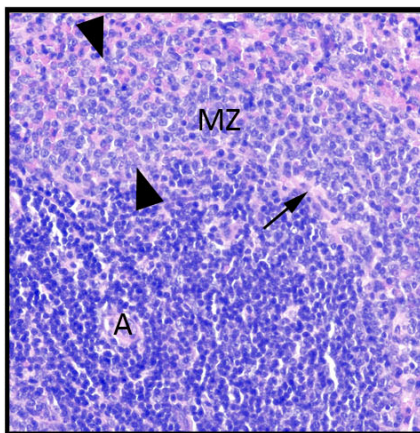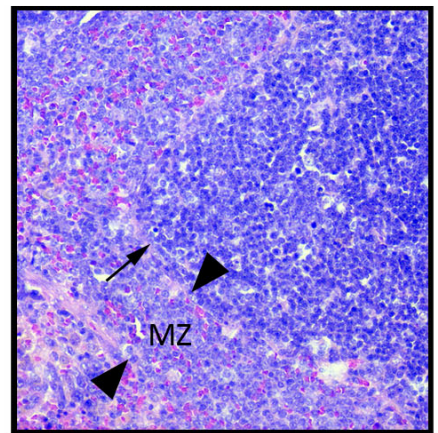

Supplement: Supplementary file 1 — We have observed macroscopically internal organs at the time of sacrifice as well as their histological sections, and concluded that there were no alterations comparing all experimental groups of animals. Specifically, in the case of the spleen, we have observed that gambogic acid treated and vehicle-treated rats showed spleen hyperplasia, with increased cellularity, compared with healthy nonarthritic rats. [file 195327.f1.pdf]
